# Supplementary material for: Altered Perception of Environmental Volatility During Social Learning in Emerging Psychosis
Source: Comput Psychiatr. 2024 Feb 7;8(1):1–22. doi: 10.5334/cpsy.95 (PMC11104374; doi:10.5334/cpsy.95)
Supplement: Supplementary material. — Supplementary simulations, supplementary results and reproducibility information. [file cpsy-8-1-95-s1.pdf]

Supplementary Material: Altered perception of environmental volatility during social learning in emerging psychosis

Daniel J. Hauke[1] Michelle Wobmann[2], Christina Andreou[3], Amatya Mackintosh[2], Renate de Bock[2], Povilas Karvelis[4]
Rick A. Adams[1,5], Philipp Sterzer[2], Stefan Borgwardt[4], Volker Roth[6], Andreea O. Diaconescu[4,7,8,9]

1 Centre for Medical Image Computing, Department of Computer Science, University College London, London, United Kingdom
2 Department of Psychiatry (UPK), University of Basel, Basel, Switzerland
3 Department of Psychiatry and Psychotherapy, Translational Psychiatry, University of Lübeck, Lübeck, Germany
4 Krembil Centre for Neuroinformatics, Centre for Addiction and Mental Health (CAMH), Toronto, Ontario, Canada
5 Max Planck Centre for Computational Psychiatry and Ageing Research, University College London, London, United Kingdom
6 Department of Mathematics and Computer Science, University of Basel, Basel, Switzerland
7 Department of Psychiatry, University of Toronto, Toronto, ON, Canada
8 Institute of Medical Sciences, University of Toronto, Toronto, ON, Canada
9 Department of Psychology, University of Toronto, Toronto, ON, Canada

Contents

1 Simulations **3**
1.1 Simulating changes in the equilibrium point 3
1.2 Simulating changes in the prior expectation about environmental volatility 4
1.3 Simulating changes in the coupling strength 5
1.4 Simulating changes in the evolution rate 6

2 Supplementary Results **7**
2.1 Behaviour between groups across experimental blocks 7
2.2 Model comparison with simpler models 8
2.3 Fixing non-recoverable parameters to test for knock-on effects 9

3 Reproducibility **12**
3.1 Behavioural results without one participant 12
3.2 Modelling results without one participant 13
3.2.1 Bayesian model selection without one participant . . . 13
3.2.2 Parameter group effects without one participant . . . 13
3.2.3 Symptom-parameter correlations without one participant 13

1 Simulations

Supplementary Figures S1 and S2 highlight the different impact that changes in the drift equilibrium point m_3 and changes in the prior expectation about environmental volatility $\mu_3^{(0)}$ have on belief trajectories at different levels of the inferential hierarchy and on simulated behavior. For completeness, we also included simulations illustrating the effects of changing the coupling between hierarchical levels κ_2 (Supplementary Figure S3) and changing the evolution rate ω_2 (Supplementary Figure S4).

1.1 Simulating changes in the equilibrium point

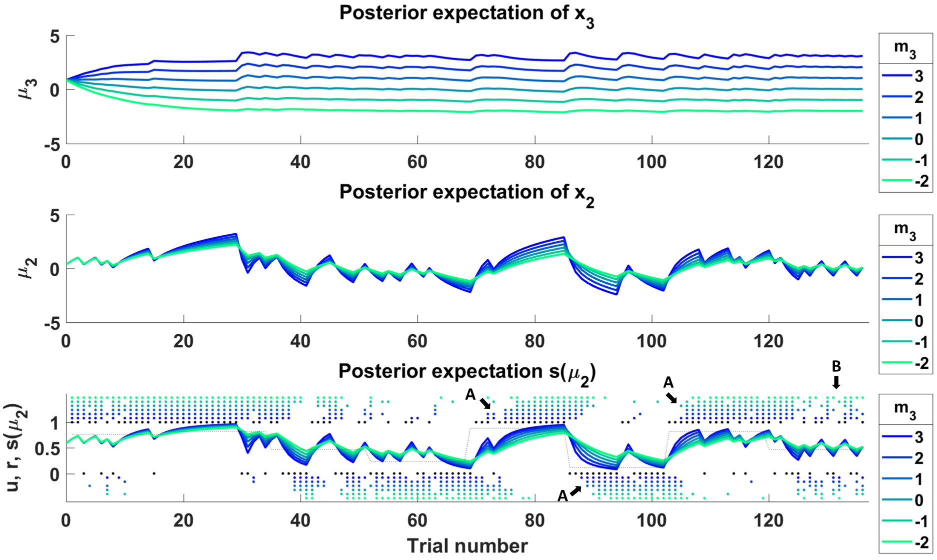

Figure S1: **Simulations showing the effect of changing the equilibrium point** m_3. Shown are trajectories of beliefs about the volatility the adviser's intentions μ_3 (upper panel), beliefs about the adviser's fidelity μ_2 (middle panel) and about the advice accuracy $s(\mu_2)$. Black dots indicate inputs (1: helpful advice, 0: misleading advice) and colored dots simulated responses (1: going with the advice, 0: going against the advice). Increasing m_3 (colder colours) results in larger precision-weighted prediction errors leading to stronger belief updates across all levels of the hierarchy that increase over the course of the session. The effect on behavior depends on the input structure. When agents are exposed to volatile changes between very helpful and very misleading advice (trials 68-119), higher m_3 leads agents to detect changes more rapidly (see black arrows labelled A). However, high values of m_3 also increase susceptibility to noisy inputs (e.g., trials 120-136; see black arrow labelled B). For the simulations, all other parameter values were fixed to the values of an ideal observer given the input.

1.2 Simulating changes in the prior expectation about environmental volatility

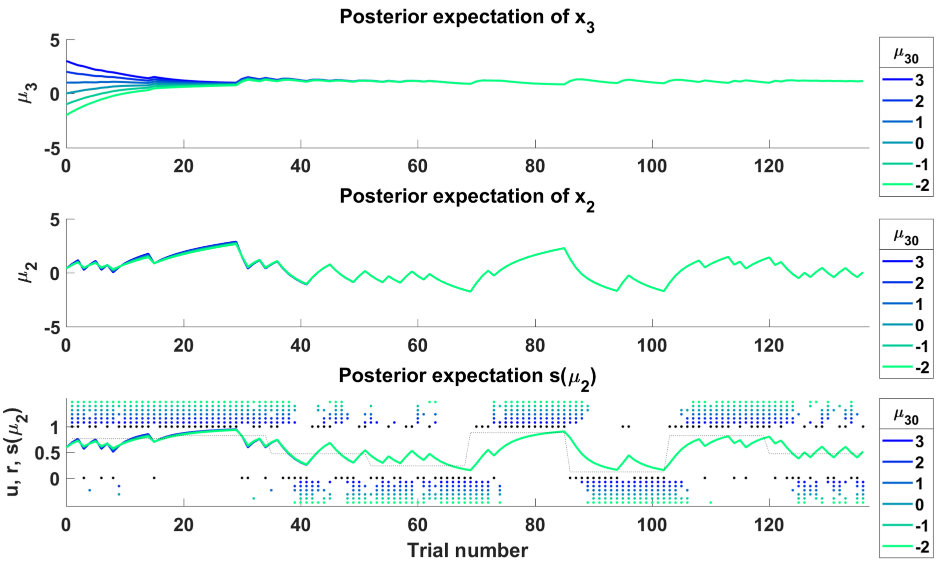

Figure S2: **Simulations showing the effect of changing the prior expectation about environmental volatility** $\mu_3^{(0)}$. Shown are trajectories of beliefs about the volatility the adviser's intentions μ_3 (upper panel), beliefs about the adviser's fidelity μ_2 (middle panel) and about the advice accuracy $s(\mu_2)$. Black dots indicate inputs (1: helpful advice, 0: misleading advice) and colored dots simulated responses (1: going with the advice, 0: going against the advice). Increasing $\mu_3^{(0)}$ (colder colours) results in changes primarily in the first trials of the session and changes do not propagate strongly to lower levels.

1.3 Simulating changes in the coupling strength

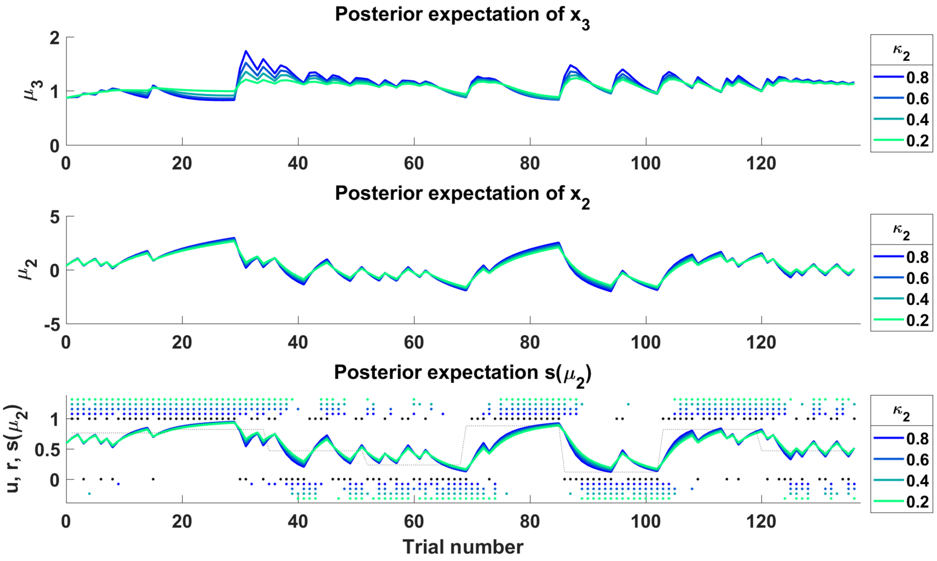

Figure S3: **Simulations showing the effect of changing the coupling strength** κ_2. Shown are trajectories of beliefs about the volatility the adviser's intentions μ_3 (upper panel), beliefs about the adviser's fidelity μ_2 (middle panel) and about the advice accuracy $s(\mu_2)$. Black dots indicate inputs (1: helpful advice, 0: misleading advice) and colored dots simulated responses (1: going with the advice, 0: going against the advice).

1.4 Simulating changes in the evolution rate

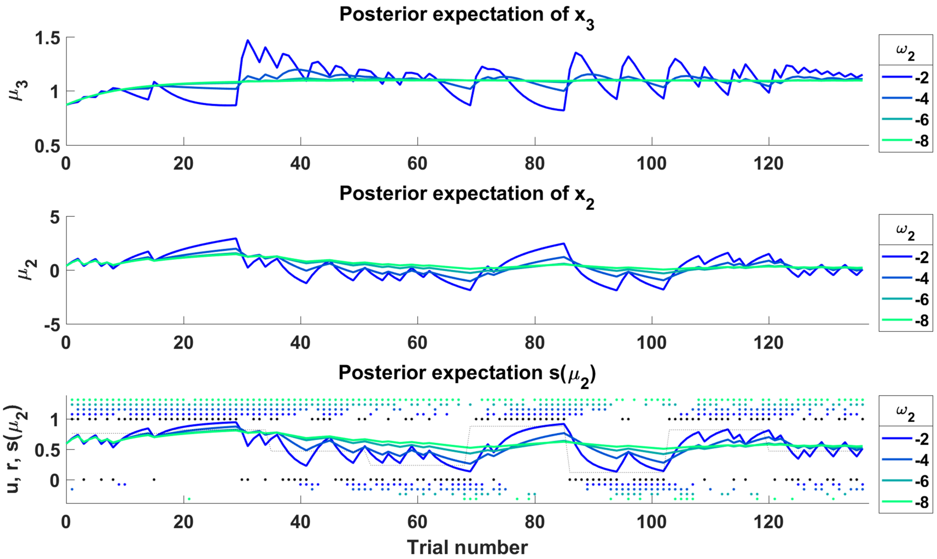

Figure S4: **Simulations showing the effect of changing the evolution rate** ω_2. Shown are trajectories of beliefs about the volatility the adviser's intentions μ_3 (upper panel), beliefs about the adviser's fidelity μ_2 (middle panel) and about the advice accuracy $s(\mu_2)$. Black dots indicate inputs (1: helpful advice, 0: misleading advice) and colored dots simulated responses (1: going with the advice, 0: going against the advice).

2 Supplementary Results

2.1 Behaviour between groups across experimental blocks

To provide a more detailed description of how the behaviour differed across groups we analysed behaviour across experimental blocks (each containing 17 trials with different advice accuracy; Figure 1B, main manuscript). We identified a significant group-by-task-block interaction on the frequency of advice-taking ($F = 3.419$, $p < 0.001$). To unpack this effect we repeated the analysis with three two-group models. We found significant group-by-task-block interactions when comparing HC vs FEP ($F = 5.416$, $p_{uncorr} < 0.001$, $p < 0.001$, Bonferroni-corrected for the number of comparisons, i.e. $n = 3$) and FEP vs CHR-P ($F = 2.616$, $p_{uncorr} = 0.013$, $p = 0.038$), but the comparison between HC vs CHR-P did not survive Bonferroni correction ($F = 2.241$, $p_{uncorr} = 0.032$, $p = 0.095$). Post hoc marginal contrasts revealed group differences in blocks 2 (HC vs FEP: $p_{uncorr} = 0.039$, $p = 0.925$), block 6 (HC vs FEP: $p_{uncorr} < 0.001$, $p = 0.004$; CHR-P vs FEP: $p_{uncorr} = 0.039$, $p = 0.929$) and block 8 (HC vs CHR-P, $p_{uncorr} = 0.042$, $p = 1.000$). However, only the effect of HC vs FEP in block 6 survived Bonferroni correction for 3 (#groups) x 8 (#blocks) = 24 comparisons, suggesting that FEP were adhering more to the advice, specifically in a block in which the adviser is truly misleading. However, note that FEP were not showing differences in helpful blocks (e.g., blocks 5 and 7), which suggests that this behaviour may not simply reflect giving up in volatile environments. Interestingly, similar findings have been reported in individuals with borderline personality disorder (Henco et al., 2020). None of the covariates significantly impacted advice taking.

The group-by-task-block interaction remained significant after including antipsychotic and antidepressant dose as covariates ($F = 3.515$, $p < 0.001$). Neither the effect of antipsychotic dose ($F = 0.447$, $p = 0.507$) or antidepressant dose ($F = 0.004$, $p = 0.950$) were significant. Unpacking this model again revealed significant group-by-task-block interactions when comparing HC vs FEP ($F = 5.416$, $p_{uncorr} < 0.001$, $p < 0.001$) and CHR-P vs FEP ($F = 3.282$, $p_{uncorr} = 0.002$, $p = 0.007$), but not when comparing HC vs CHR-P ($F = 1.452$, $p_{uncorr} = 0.185$, $p = 1.00$). The group-by-task-phase interaction effect in HC vs CHR-P did not survive Bonferroni correction ($F = 5.154$, $p_{uncorr} = 0.030$, $p = 0.556$). Post hoc marginal contrasts revealed group differences in block 6 (HC vs FEP: $p_{uncorr} < 0.001$, $p = 0.005$; CHR-P vs FEP: $p_{uncorr} = 0.014$, $p = 0.348$; Figure SS5) and block 8 (HC vs CHR-P, $p_{uncorr} = 0.038$, $p = 0.904$). However, only the effect of HC vs FEP in block 6 survived Bonferroni correction for 3 (#groups) x 8 (#blocks) = 24 comparisons.

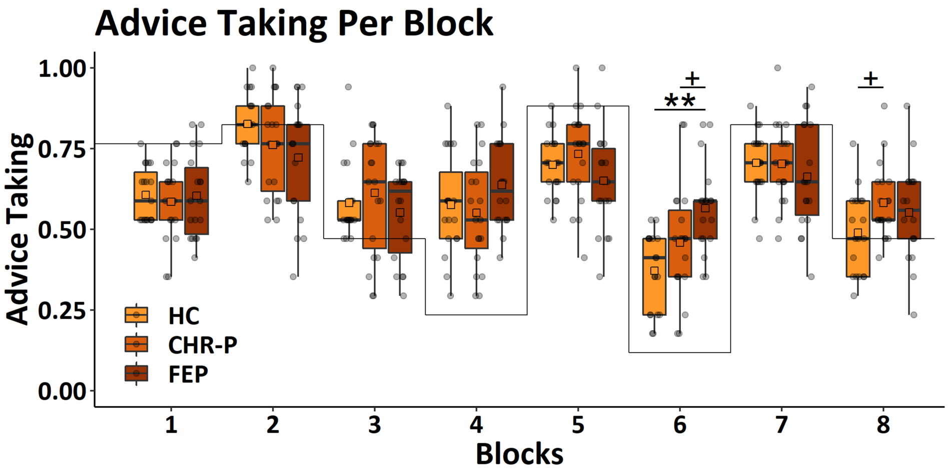

Figure S5: **Behaviour between groups and across experimental block**. The black line indicates the average advice accuracy of each block. Horizontal lines and squares in box plots represent median and mean, respectively. Boxes span the 25th to 75th quartiles and whiskers extend from hinges to the largest and smallest value that lies within 1.5× interquartile range. Asterisks indicate significance of marginal tests at: ** $p < 0.01$, using Bonferroni correction, adjusted for 3 (#groups) x 8 (#blocks) = 24 comparisons, or at $^+$ $p < 0.05$ uncorrected.

2.2 Model comparison with simpler models

To test whether simpler models were sufficient to explain participants' behaviour in this social learning task, we conducted a supplementary analysis in which we added two simpler models to the model space, a Rescorla-Wagner model and a non-hierarchical two-level HGF. The priors for these models are reported in Table SS1. Model comparison favoured the models reported in the main manuscript (Figure SS6) in line with previous model comparisons (Cole et al., 2020; Diaconescu et al., 2014, 2017, 2020)).

Rescorla-Wagner

Parameter	Model Component	Prior Mean	Prior Variance	Transformation	Bounds	Fixed?	Based on
α	Perceptual Model	0.25[a]	1[a,b]	logit	[0, 1]	-	
$\upsilon^{(0)}$	Perceptual Model	0.5[a,b]	1[a,b]	logit	[0, 1]	-	
ν	Response Model	48[a,b]	1[a,b]	log	$[0, +\infty)$	-	

2-level HGF

Parameter	Model Component	Prior Mean	Prior Variance	Transformation	Bounds	Fixed?	Based on
ω_2	Perceptual Model	-2[b,c]	4[c]	-	$(-\infty, +\infty)$	-	
κ_2	Perceptual Model	0[a]	0[a]	logit	[0, 1]	Yes	[a]
$\sigma_2^{(0)}$	Perceptual Model	0[a,b]	0[a]	-	$(-\infty, +\infty)$	Yes	[a]
$\sigma_2^{(0)}$	Perceptual Model	1[a,b]	0[a]	log	$[0, +\infty)$	Yes	[a]
ν	Response Model	48[a,b,c]	1[a,b,c]	log	$[0, +\infty)$	-	

Table S1: **Model parameter overview for simpler models**. Prior parameter values were chosen based on the references indicated next to prior means and variances. [a]Cole et al. (2020). [a]Cole et al. (2020). [b]Diaconescu et al. (2014). [c]Hauke et al. (2018).

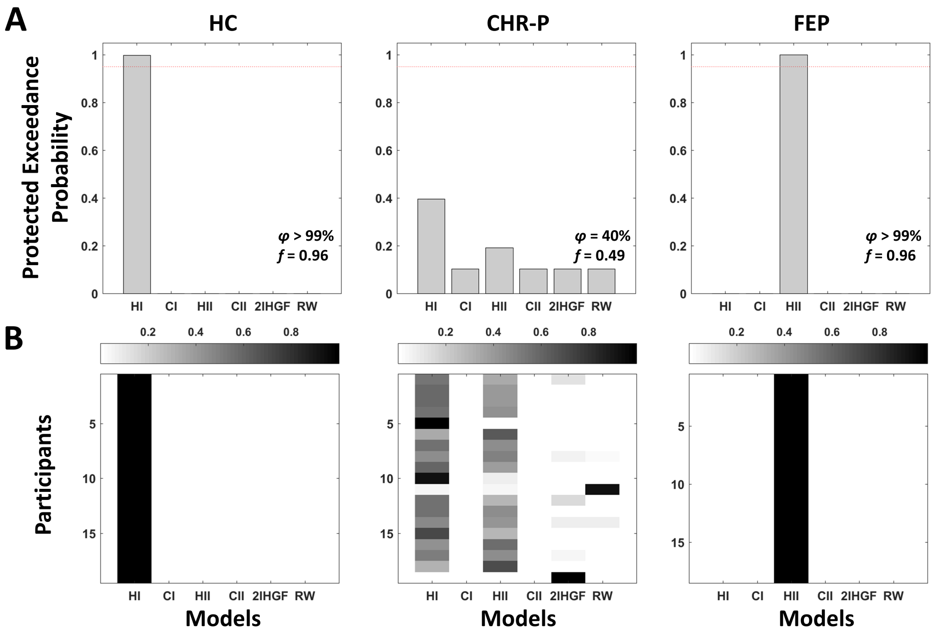

Figure S6: **Bayesian model selection results with simpler models**. **A** Protected exceedance probabilities for within-group random-effects Bayesian model selection (Stephan et al., 2009; Rigoux et al., 2014) to arbitrate between Hypothesis I (**HI**; standard 3-level HGF) and Hypothesis II (**HII**; mean-reverting HGF with drift at 3rd level in line with an altered perception of volatility). Two corresponding control models were included (**CI** and **CII**), for which the perceptual model parameters were fixed. Additionally, two simpler models, a Rescorla-Wagner model (**RW**) and a 2-level HGF (**2lHGF**), were included in this extended comparison. Model selection was performed separately in healthy controls (**HC**), individuals at clinical high risk for psychosis (**CHR-P**), or first-episode psychosis patients (**FEP**). The dashed line indicates 95% exceedance probability. **B** Model attributions for each participant.

2.3 Fixing non-recoverable parameters to test for knock-on effects

To assess whether non-recoverable parameters led to knock-on effects on other parameters, we repeated the analyses presented in the main manuscript, while fixing parameters that could not be recovered in the main manuscript to their priors (i.e., $\mu_2^{(0)} = 0$, $\mu_3^{(0)} = 1$, and $\kappa_2 = 0.5$). We found that the main result (Bayesian model selection) was comparable when fixing non-recoverable parameters (Figure SS7).

When repeating the secondary analysis investigating parameter group effects on m_3 to assess whether psychosis was associated with perceiving the environment as increasingly volatile or stable, we again found an effect on m_3 ($\eta^2 = 0.111$, $p_{uncorr} = 0.048$; Figure SS8). As in the main manuscript, this effect did not survive Bonferroni correction for the number of free parameters ($p = 0.191$). Pairwise comparison revealed higher m_3 in CHR vs HC ($p_{uncorr} = 0.021$) and a trend-effect of higher m_3 FEP vs HC ($p_{uncorr} = 0.060$). Both did not survive Bonferroni correction for $n = 3$ groups ($p = 0.063$ and $p = 0179$).

Lastly, we found trend correlations between m_3 and PANSS positive symptoms ($\tau = 0.185$, $p_{uncorr} = 0.058$ and $p = 0.175$, adjusted for $n = 3$ PANSS scales; Figure SS8) and between m_3 and PCL frequency scores ($\tau = 0.163$, $p_{uncorr} = 0.084$ and $p = 0.253$, adjusted for $n = 3$ PCL scales). As stated in the main manuscript, although these correlations were in the expected direction, they should be assessed in a larger sample as our current results are inconclusive.

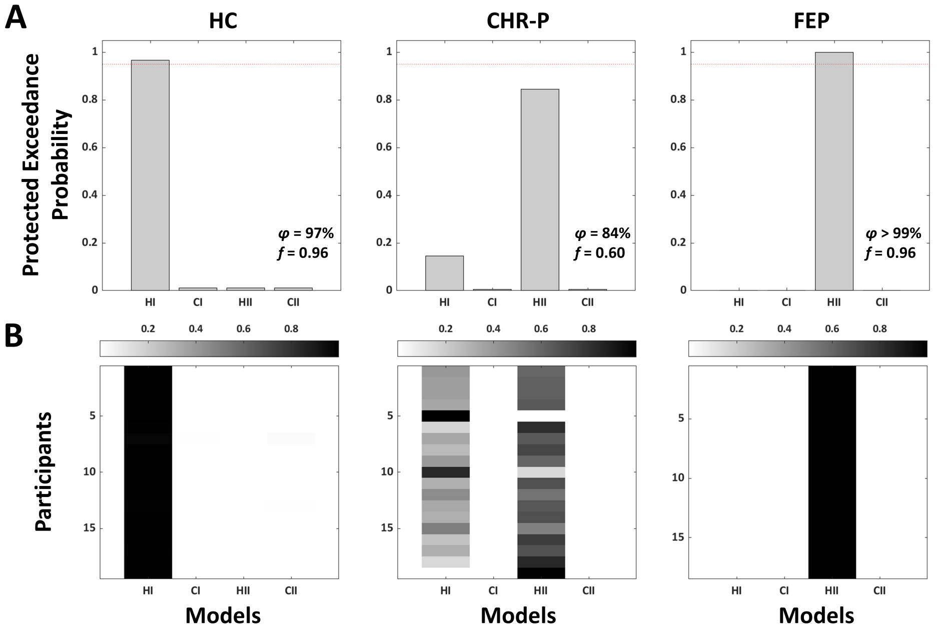

Figure S7: **Bayesian model selection results with fixed non-recoverable parameters**. **A** Protected exceedance probabilities for within-group random-effects Bayesian model selection (Stephan et al., 2009; Rigoux et al., 2014) to arbitrate between Hypothesis I (**HI**; standard 3-level HGF) and Hypothesis II (**HII**; mean-reverting HGF with drift at 3rd level in line with an altered perception of volatility). Two corresponding control models were included (**CI** and **CII**), for which the perceptual model parameters were fixed. Model selection was performed separately in healthy controls (**HC**), individuals at clinical high risk for psychosis (**CHR-P**), or first-episode psychosis patients (**FEP**). The dashed line indicates 95% exceedance probability. **B** Model attributions for each participant.

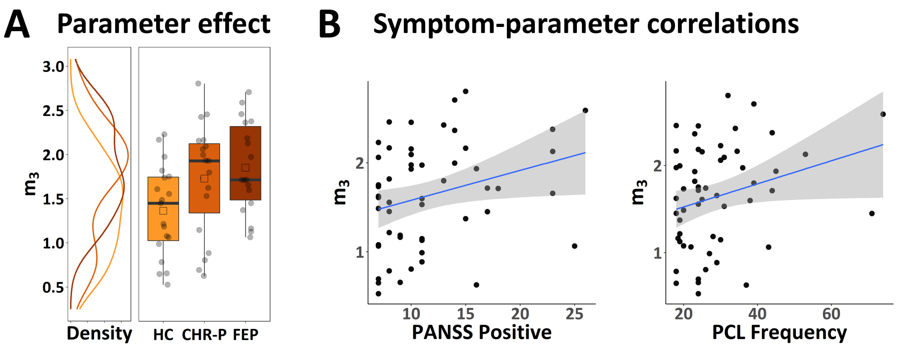

Figure S8: **Parameter group effects with fixed non-recoverable parameter**. **A** Parameter effect for drift equilibrium point m_3. **B** Correlation between model parameters and either Positive and Negative Syndrome Scale (Kay et al., 1987) (**PANSS**) or Paranoia Checklist (Freeman et al., 2005) (**PCL**). Note, that raw scores are displayed for illustration purposes only. Statistical analyses were conducted using nonparametric Kendall rank correlations. Displayed regression lines were computed using a linear model based on the raw scores. **P**: Positive symptoms. Boxes span the 25th to 75th quartiles and whiskers extend from hinges to the largest and smallest value that lies within 1.5× interquartile range.

3 Reproducibility

To ensure reproducibility of our results, we report the results excluding one participant who did not consent to make their data available for reuse and was excluded from the public repository. Our entire analysis pipeline with instructions can be found here: `https://github.com/daniel-hauke/compi_ioio_phase`. The data can be downloaded here: `https://osf.io/6rdjc/`.

The models were implemented in Matlab (version: 2017a; `https://mathworks.com`) using the HGF toolbox (version: 3.0), which is made available as open-source code as part of the TAPAS software collection (`https://github.com/translationalneuromodeling/tapas/releases/tag/v3.0.0`; Frässle et al. (2021)) and the VBA toolbox (Daunizeau et al., 2014) (`https://mbb-team.github.io/VBA-toolbox/`; Daunizeau et al. (2014)). Statistical analyses were run in R (version: 4.04; `https://www.r-project.org/`) using R-Studio (version: 1.4.1106; `https://www.rstudio.com/`).

3.1 Behavioural results without one participant

Excluding the participant who did not consent to reusing their data, we still identified a significant group-by-task-phase interaction on the frequency of advice-taking ($F = 4.857$, $p = 0.012$). To unpack this effect we repeated the analysis with three two-group models. We found significant group-by-task-phase interactions when comparing HC vs FEP ($F = 7.128$, $p_{uncorr} = 0.012$, $p = 0.035$ Bonferroni-corrected for the number of comparisons, i.e. $n = 3$) and HC vs CHR-P ($F = 7.745$, $p_{uncorr} = 0.009$, $p = 0.026$), but not when comparing CHR-P vs FEP ($F = 0.001$, $p_{uncorr} = 0.977$, $p = 1.000$), suggesting that both CHR-P and FEP showed reduced flexibility to take environmental volatility into account as the difference between stable and volatile phase was reduced compared to HC. None of the covariates significantly impacted advice taking.

The group-by-task-phase interaction remained significant after including antipsychotic and antidepressant dose as covariates ($F = 4.296$, $p = 0.019$). Neither the effect of antipsychotic dose ($F = 0.240$, $p = 0.627$) or antidepressant dose ($F = 0.096$, $p = 0.759$) were significant. Unpacking this model again revealed significant group-by-task-phase interactions when comparing HC vs FEP ($F = 7.128$, $p_{uncorr} = 0.012$, $p = 0.035$), but not when comparing CHR-P vs FEP ($F = 0.322$, $p_{uncorr} = 0.574$, $p = 1.00$). The group-by-task-phase interaction effect for HC vs CHR-P did not survive Bonferroni correction ($F = 5.154$, $p_{uncorr} = 0.030$, $p = 0.089$).

3.2 Modelling results without one participant

3.2.1 Bayesian model selection without one participant

We also repeated the Bayesian model selection including participants from all groups first. The results were again inconclusive (ϕ = 59.92%, f = 51.22% in favour of Hypothesis II) possibly suggesting that different groups were best explained by different models (i.e., different computational mechanisms). To assess this possibility, we repeated the model selection for each group separately. In HC, the winning model was the standard 3-level HGF (Hypothesis I; ϕ = 96.63%, f = 95.93%). Conversely, in FEP the mean-reverting HGF that included a drift at the third level was selected (Hypothesis II; ϕ = 99.89%, f = 95.69%). For CHR-P, we observed a more heterogeneous results: While the mean-reverting model was favoured (Hypothesis II; ϕ = 84.50%, f = 60.24%), there was also evidence for the standard HGF, albeit to a much lesser extent (Hypothesis I; ϕ = 14.41%, f = 37.19%). Further inspection of the model attributions for all individual participants revealed an interesting pattern. All HC were attributed to the standard HGF with over 97% probability, whereas FEP were attributed to the mean-reverting model with over 99%. Interestingly, model attributions for CHR-P were more heterogeneous ranging from 0 to 100% probability, suggesting that some individuals were better explained by the standard HGF, but others by the mean-reverting model.

3.2.2 Parameter group effects without one participant

In the reduced sample, the drift equilibrium point m_3 significantly differed across the groups ($\eta^2 = 0.130, p_{uncorr} = 0.030$). Post hoc tests revealed that m_3 was increased in FEP compared to HC suggesting that FEP perceived the intentions of the adviser as increasingly more volatile over time ($\eta^2 = 0.191, p = 0.029$, Bonferroni-corrected for the number of comparisons across groups, i.e., $n = 3$). We also performed an exploratory analysis including all other free model parameters. This analysis revealed an additional effect on coupling strength κ_2 ($\eta^2 = 0.157, p_{uncorr} = 0.014$), which was driven by reduced coupling strength between the second and third level of the perceptual hierarchy in FEP compared to HC ($\eta^2 = 0.245, p = 0.010$, Bonferroni-corrected for the number of comparisons across groups, i.e., $n = 3$). However, neither the effect on m_3 nor κ_2 survived Bonferroni correction for the number of parameters, i.e. $n = 7$ ($p = 0.207$ and $p = 0.100$, respectively), possibly due to a lack of power.

3.2.3 Symptom-parameter correlations without one participant

Repeating the correlations on the reduced sample yielded a positive trend correlation between m_3 and PANSS positive symptoms ($\tau = 0.175$, $p_{uncorr} =$

0.077, p = 0.460 Bonferroni-adjusted for 2 (#parameters) x 3 (#PANSS subscales) = 6 comparisons). As in the main manuscript, there were negative correlations between κ_2 and PANSS negative and general symptoms ($\tau = -0.265$, $p_{uncorr} = 0.008$, $p = 0.052$ and $\tau = -0.24$, $p_{uncorr} = 0.013$, $p = 0.077$ respectively), which did not survive Bonferroni correction.

Similarly, there was only a trend correlation when excluding this subject between m_3 and the PCL frequency subscale ($\tau = 0.170$, $p_{uncorr} = 0.079$, $p = 0.475$ Bonferroni-adjusted for 2 (#parameters) x 3 (#PCL subscales) = 6 comparisons). Note, that the participant that did not consent to reuse of their data scored high on positive symptoms which likely contributes to the changes in the correlations compared to the full sample. However, as we pointed out in the main manuscript these correlation results should be taken as preliminary due to the small sample size of this study.

References

Cole, D. M., Diaconescu, A. O., Pfeiffer, U. J., Brodersen, K. H., Mathys, C. D., Julkowski, D., Ruhrmann, S., Schilbach, L., Tittgemeyer, M., Vogeley, K., et al. (2020). Atypical processing of uncertainty in individuals at risk for psychosis. *NeuroImage: Clinical*, 26:102239.

Daunizeau, J., Adam, V., and Rigoux, L. (2014). VBA: a probabilistic treatment of nonlinear models for neurobiological and behavioural data. *PLoS computational biology*, 10(1):e1003441.

Diaconescu, A. O., Mathys, C., Weber, L. A., Daunizeau, J., Kasper, L., Lomakina, E. I., Fehr, E., and Stephan, K. E. (2014). Inferring on the intentions of others by hierarchical Bayesian learning. *PLoS Computational Biology*, 10(9):e1003810.

Diaconescu, A. O., Mathys, C., Weber, L. A., Kasper, L., Mauer, J., and Stephan, K. E. (2017). Hierarchical prediction errors in midbrain and septum during social learning. *Social Cognitive and Affective Neuroscience*, 12(4):618–634.

Diaconescu, A. O., Wellstein, K. V., Kasper, L., Mathys, C., and Stephan, K. E. (2020). Hierarchical Bayesian models of social inference for probing persecutory delusional ideation. *Journal of Abnormal Psychology*, 129(6):556–569.

Frässle, S., Aponte, E. A., Bollmann, S., Brodersen, K. H., Do, C. T., Harrison, O. K., Harrison, S. J., Heinzle, J., Iglesias, S., Kasper, L., et al. (2021). TAPAS: an open-source software package for translational neuromodeling and computational psychiatry. *Frontiers in Psychiatry*, 12:857.

Freeman, D., Garety, P. A., Bebbington, P. E., Smith, B., Rollinson, R., Fowler, D., Kuipers, E., Ray, K., and Dunn, G. (2005). Psychological investigation of the structure of paranoia in a non-clinical population. *The British Journal of Psychiatry*, 186(5):427–435.

Hauke, D. J., Diaconescu, A. O., Wellstein, K., Tomiello, S., Rigoux, L., Heinzle, J., and Stephan, K. E. (2018). F237. dopaminergic effects on hierarchical prediction errors and connectivity during social learning. *Schizophrenia Bulletin*, 44(suppl_1):S314–S315.

Henco, L., Diaconescu, A. O., Lahnakoski, J. M., Brandi, M.-L., Hörmann, S., Hennings, J., Hasan, A., Papazova, I., Strube, W., Bolis, D., et al. (2020). Aberrant computational mechanisms of social learning and decision-making in schizophrenia and borderline personality disorder. *PLoS Computational Biology*, 16(9):e1008162.

Kay, S. R., Fiszbein, A., and Opler, L. A. (1987). The Positive and Negative Syndrome Scale (PANSS) for schizophrenia. *Schizophrenia Bulletin*, 13(2):261–276.

Rigoux, L., Stephan, K., Friston, K., and Daunizeau, J. (2014). Bayesian model selection for group studies — revisited. *NeuroImage*, 84:971–985.

Stephan, K. E., Penny, W. D., Daunizeau, J., Moran, R. J., and Friston, K. J. (2009). Bayesian model selection for group studies. *NeuroImage*, 46(4):1004–1017.
